# Dendritic Cells Are Critical for the Activation and Expansion of Vδ2^+^ T Cells After Allogeneic Hematopoietic Transplantation

**DOI:** 10.3389/fimmu.2018.02528

**Published:** 2018-11-01

**Authors:** Xiaoyu Wang, Jiangying Liu, Haitao Gao, Xiao-Dong Mo, Tingting Han, Lan-Ping Xu, Xiao-Hui Zhang, Xiao-Jun Huang

**Affiliations:** ^1^Peking University People's Hospital, Peking University Institute of Hematology, Beijing Key Laboratory of Hematopoietic Stem Cell Transplantation, Beijing, China; ^2^Beijing Hightrust Diagnostics, Co., Ltd, Beijing, China

**Keywords:** hematopoietic stem cell transplantation, immune reconstitution, γδ T cells, dendritic cells, aminobisphosphonate

## Abstract

γδ T cells perform antitumor and antiviral effector functions and are involved in both innate and adaptive immunity. Vδ2^+^ T cells represent the predominant γδ T subset in the peripheral blood of healthy subjects. Vδ2^+^ T cells can be selectively activated and expanded by phosphoantigens (pAgs). Dendritic cells (DCs), as potent antigen-presenting cells, are capable of mediating pAgs–triggered Vδ2^+^ T cells expansion. However, the association between DCs and Vδ2^+^ T cell recovery in the context of hematopoietic stem cell transplantation (HSCT) remains unclear. We previously demonstrated that the recovery of Vδ2^+^ T cells was hampered and inversely correlated with Epstein-Barr virus (EBV) reactivation in patients undergoing haploidentical HSCT (haploHSCT). Whether Vδ2^+^ T cells from haploHSCT recipients can be expanded by stimulation with aminobisphosphonates or pAg–presenting DCs is of particular interest. Herein, we showed that Vδ2^+^ T cells recovered after haploHSCT failed to expand after *ex-vivo* stimulation with pamidronate. In addition, we found that the recovery of DC subsets was significantly decreased, and the concentration of myeloid DCs (mDCs) correlated significantly with Vδ2^+^ T cell recovery in the setting of allogeneic HSCT. Furthermore, coculture of peripheral lymphocytes from recipients with monocyte-derived and pamidronate-pretreated autologous or allogeneic DCs induced the successful expansion of Vδ2^+^ T cells. Of note, allogeneic DCs from third-party donors stimulated a significantly higher efficiency of Vδ2^+^ T cell expansion than autologous DCs. More importantly, the memory features were well-retained and the cytotoxic cytokines-production capacity was significantly enhanced in the expanded Vδ2^+^ T cells. Taken together, these results suggest that the frequency and function of DCs are critical for the recovery of Vδ2^+^ T cells after allogeneic HSCT. The fact that vigorous expansions of Vδ2^+^ T cells were induced by phosphoantigen-pretreated DCs, especially by allogeneic third-party DCs, provides additional options for the development of individualized immunotherapy strategies that utilize the anti-viral and anti-leukemic effects of γδ T cells in the context of hematopoietic transplantation.

## Introduction

Immune reconstruction is critical for the therapeutic efficacy and outcomes of patients who undergo hematopoietic stem cell transplantation (HSCT) (1, 2). Although most studies in the literature have focused on the recovery characteristics and functional features of either adaptive cytotoxic CD8^+^ T cells or natural killer cells (3–5), γδ T cells, which have innate-like cytotoxicity, are increasingly recognized as promising immune effector cells in the context of HSCT (6, 7). Improved disease-free survival was shown in HSCT recipients with increased γδ T-cell counts compared to those with normal/decreased γδ T numbers (8). Perko et al. reported that an elevated number of γδ T cells was significantly associated with a lower probability of infections in children who underwent allogeneic HSCT (9). In humans, γδ T cells comprise ~1–10% of peripheral blood T lymphocytes. The dominant subset of circulating γδ T cells expresses the Vδ2 T cell receptor (TCR) paired with the Vγ9 TCR (10, 11). Our study recently demonstrated that the recovery of Vδ2^+^ T cells inversely correlated with Epstein-Barr virus (EBV) reactivation in adult recipients following haploidentical HSCT (haploHSCT). Notably, the frequencies of Vδ2^+^ T cells, in contrast to Vδ2-negative cells, generally decreased from 30 to 90 days after transplantation, regardless of the status of EBV reactivation (12). It is worth exploring whether this phenomenon still exists at later stages after transplantation. The factors associated with the impairment of Vδ2^+^ T cells recovery and strategies for restoring this special T subset have not been thoroughly investigated.

Vδ2^+^ T cells are specifically activated and expanded by small non-peptidic phosphoantigens (pAgs), which are intermediates in the microbial and eukaryotic isoprenoid biosynthesis pathway (13, 14). Aminobisphosphonates, such as pamidronate and zoledronate, can inhibit the mevalonate pathway and result in the intracellular accumulation of isopentenyl pyrophosphate (IPP), which selectively stimulates Vδ2^+^ T cells *in vitro* and *in vivo* (15, 16). More recently, evidences highlighted the butyrophilin family member BTN3A1 (CD277), a glycoprotein that acts as a sensor in mediating pAg-induced Vδ2^+^ T cell proliferation. The binding of isoprenoid metabolites to the intracellular domain of CD277, B30.2, can be recognized by the Vδ2 TCR, which leads to the functional activation of Vδ2^+^ T cells (17–19). In addition, dendritic cells (DCs), as the most potent antigen-presenting cells, have been reported to stimulate γδ T cell proliferation by presenting pAgs through CD277. Several studies have shown that aminobisphosphonate-treated DCs can stimulate the strong expansion of Vδ2^+^ T cells with high cytotoxic activity from healthy donors (20–23). Although some protocols for adoptive immunotherapy using aminobisphosphonate or aminobisphosphonate-pretreated DCs have yield the successful expansion of Vδ2^+^ T cells in healthy subjects and patients with solid tumors or hematologic malignancies (21, 24–26), very few studies have transferred these strategies to the context of HSCT. Airoldi et al. and Bertaina et al. reported that peripheral Vδ2^+^ T cells from pediatric patients who received haploHSCT with the depletion of CD19^+^ B cells and αβ^+^ T cells, were efficiently expanded upon exposure to zoledronate (27, 28). However, the correlation of DC concentrations with Vδ2^+^ T cell recovery in the context of HSCT remains unknown. Following the wide use of unmanipulated haploHSCT for the treatment of hematopoietic disease, whether aminobisphosphonate or aminobisphosphonate-pretreated DCs promote Vδ2^+^ T cell activation in this setting is of interest.

In the present study, we investigated the influences of DCs on the *in vivo* recovery and *ex-vivo* expansion of Vδ2^+^ T cells after hematopoietic transplantation. In light of the observation that there is a significant correlation of DCs content with Vδ2^+^ T cells recovery, we attempted to utilize pamidronate-pretreated autologous or allogeneic third-party DCs to restore the expansion of Vδ2^+^ T cells in HSCT recipients.

## Materials and methods

### Patients

To evaluate the levels of reconstituted Vδ2^+^ T cells and DCs, 35 consecutive adult patients with hematopoietic malignancies and received haploHSCT at Peking University People's Hospital were included from April 2017 to June 2017. Peripheral blood samples of 20 healthy donors were collected as controls from routine clinical examination procedures. Protocol of study has been approved by the Ethics Committee of Peking University Institute of Hematology. All recipients and donors signed consent forms.

### Flow cytometry

Immunophenotyping analyses for the recovered Vδ2^+^ T cells and DCs were performed with flow cytometry ~180 days post-haploHSCT. Briefly, fresh peripheral blood cells were stained with the following fluorochrome-labeled antibodies: PE-Cy7 anti-CD3, BV421 anti-TCRγδ, Alexa Fluor700 anti-TCRVδ2, FITC anti-Lineage Cocktail (CD3/14/19/56), PE/Dazzle 594 anti-HLA-DR, BV711 anti-CD11c, APC anti-CD123, and PE anti-CD277 were purchased from BioLegend (San Diego, CA, USA). Polychromatic flow cytometric analyses were performed on a BD LSRFortessaTM Cell Analyser and further analyzed using BD FACSDiva^TM^ software.

### RNA isolation, cDNA synthesis, and real time PCR

γδ T cells were isolated from peripheral blood mononuclear cells (PBMCs) by magnetic bead separation using the Anti-TCR γ/δ MicroBead Kit (Miltenyi Biotec, Bergisch Gladbach, Germany). The purified γδ T cells were harvested and total RNA was extracted by RNA Cell Miniprep System according to the manufacturer's protocol (Promega, USA). The cDNA was synthesized with Oligo(dT)_18_ primer and Superscript II Reverse Transcriptase (Invitrogen, USA). The mRNA levels were measured by quantitative PCR using ABI PRISM 7700 Sequence Detection System (Applied Biosystems). *ABL* was used as internal reference gene and the expression levels of *CD277* and *B30.2* were represented by the relative percentages compared with those of the *ABL* (ratio = 2^−Δ*Ct*^ × 100%).

Primer sequence information is as follows:

*CD277*-forward: 5′-CGGGGAGAGAGACATTCAGC-3′;

*CD277*-reverse: 5′-AAGGAGGATGGGGTTTGCTG-3′;

*B30.2*-forward: 5′-GGAGGTAGGGGACAGGAAAG-3′;

*B30.2*-reverse: 5′-CCATCAGTCAGCCCCATAGT-3′;

*ABL*-forward: 5′-TGGAGATAACACTCTAAGCATAACTAAAGGT-3′;

*ABL*-reverse: 5′-GATGTAGTTGCTTGGGACCCA-3′.

### *Ex-vivo* expansion of Vδ2^+^ T cells by pamidronate

PBMCs were isolated from blood samples of healthy donors or HSCT recipients by Ficoll-Hypaque density gradient centrifugation, and cultured in RPMI 1640 medium supplemented with 10% FBS. Pamidronate was added at day 0 and day 3 to a final concentration 9 μg/ml. Recombinant human interleukin-2 (Invitrogen, Carlsbad, CA, USA) was added to a final concentration of 500 IU/ml every third day from day 3. After 7 and 14 days of culture, cells were harvested and stained with fluorochrome-labeled anti-CD3, anti-γδT, and anti-TCRVδ2 antibodies, and the percentages of Vδ2^+^ T cells were measured by flow cytometry.

### Generation of monocytes-derived dendritic cells

PBMCs from healthy donors or HSCT recipients were positively selected using CD14-targeting microBeads according to the manufacturer's instructions (Miltenyi Biotec, Bergisch Gladbach, Germany). Then DCs were induced from CD14^+^ monocytes as previously described with some minor modifications (20). Briefly, CD14^+^ cells were seeded in 24-well plates at 1 × 10^6^ cells/ml in RPMI-1640 medium supplemented with 10% FBS, 1,000 U/ml GM-CSF, and 500 U/ml IL-4 for 5 days. On day 6, immature DCs were collected and induced for maturation with 10 ng/ml of IL-1β, 1,000 IU/ml of TNFα, 10 ng/ml of IL-6 (purchased from Stemimmune LLC, USA), and 1 μg/ml of LPS (Sigma Aldrich, USA), in the presence of 9 μg/ml of pamidronate, for an additional 48 h.

### Co-culture of Vδ2^+^ T cells with pamidronate-pretreated DCs

Peripheral blood lymphocytes (PBLs) from HSCT recipients were cocultured with pamidronate-pretreated autologous or allogeneic (refers to third-party healthy subjects) DCs at the ratio of 2:1, at a final of 3 × 10^5^ cells/well in 96-well plates. In some experiments, the neutralization antibody anti-NKG2D (10 μg/ml, BioLegend, USA) was used for blocking NKG2D during coculture. After 7 and 14 days of coculture, the percentages and differentiation and activation phenotypes of Vδ2^+^ T cells were detected by flow cytometry. Different combinations of monoclonal antibodies allowed identifying the differentiation profile of Vδ2^+^ T cells by the expression of CD45RO and CD27 (Naive: CD45RO^−^CD27^+^, Central Memory (CM): CD45RO^+^CD27^+^, Effector Memory (EM): CD45RO^+^CD27^−^ and terminal differentiation (TD): CD45RO^−^CD27^−^). For cell differentiation and activation assay, the cultured cells were stained with fluorochrome-labeled anti-CD27, anti-CD45RO, anti-HLA-DR, anti-CD38, and anti-NKG2D antibodies (BioLegend, USA).

### Intracellular staining of Ki67, IFN-γ, and TNF-α

PBLs without treatment, PBLs stimulated with pamidronate, and PBLs cocultured with auto- or allo- DCs^pami+^ were treated with phorbol myristate acetate (PMA) and ionomycin Cocktail (Thermo Fisher Scientific) at 37°C for 4 h. Cells were collected, fixed and permeabilized with the FIX&PERM kit (MultiSciences Biotech, China) according to the manufacturer's instructions, and stained with PE anti-ki67, BV421 anti-IFN-γ, and PerCP anti-TNF-α antibodies (Biolegend, USA).

### Cytotoxicity assay of DCs^pami+^-expanded Vδ2^+^ T cells

After 14 days of coculture, Vδ2^+^ T cells expanded by alloDCs^pami+^ were isolated using the Anti-TCR γ/δ MicroBead Kit. The purity of Vδ2 cells was detected by flow cytometry (all ≥90%). The purified Vδ2 cells were cocultured with K562 cells at 5:1 ratio for 4 h. Then cells were stained with the fluorochrome-labeled anti-CD3 and anti-TCR Vδ2 antibodies as described above. For cell apoptosis assay, cells were resuspended in AnnexinV Binding Buffer (BioLegend) with APC-conjugated AnnexinV (BioLegend). Polychromatic flow cytometric analyses were performed on a BD LSRFortessaTM Cell Analyser and further analyzed using BD FACSDivaTM software.

### Statistical analysis

Statistical analyses were performed using SPSS 22.0 statistical software (SPSS Inc, Chicago, IL, USA). Differences in age and proportions of reconstituted Vδ2^+^ T cells and DCs between recipients and healthy donors were compared using the Mann–Whitney test. Difference in the proportions of gender was analyzed using the Chi-squared test. Bivariate correlation of the recoveries between peripheral Vδ2^+^ T cells and DCs was analyzed using the Spearman test. The differences in expansion proportions of Vδ2^+^ T cells and cytokine-expressing cells among groups with different stimulations were compared using the One-Way ANOVA analysis. Statistical significance was defined as *P* ≤ 0.05, based on a two-tailed test.

## Results

### The frequency of Vδ2^+^ T cells was significantly decreased after HaploHSCT and failed to increase after *ex-vivo* stimulation with pamidronate

**Figure 1:**
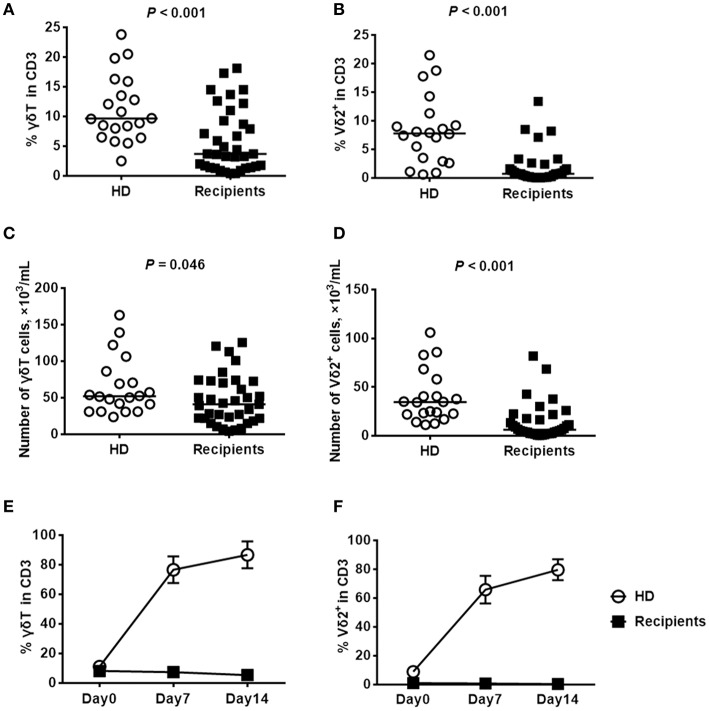
Comparisons of the proportions of γδ T cell subsets and the *ex-vivo* expansions of Vδ2^+^ T cells in response to pamidronate between haploHSCT recipients and healthy donors. The proportions and absolute numbers of γδ T cells **(A,C)** and Vδ2^+^ T cells **(B,D)** among the total CD3^+^ cells were detected by flow cytometry in recipients 180 days after haploHSCT (*n* = 35) or in healthy donors (*n* = 20). Expansion of γδ T cells **(E)** and Vδ2^+^ T cells **(F)** from haploHSCT recipients and healthy donors after treatment with pamidronate for 7 and 14 days, respectively. *P*-values are shown on the graphs, *n* = 5.

Our previous studies demonstrated that the recovery of Vδ2^+^ T cells was continuously delayed in recipients at 30, 60, and 90 days after haploHSCT (12, 29). Herein, we detected the concentration of Vδ2^+^ T cells over a longer time course (180 days) after transplantation, in comparison with healthy donors. The clinical characteristics of patients are summarized in Table 1, and there were no significant differences in the median age or the gender ratio between the included recipients and healthy donors. At the time of sampling, the included patients were all negative for cytomegalovirus (CMV) and EBV DNA in the peripheral blood. As shown in Figure 1, compared with those of the healthy donors, the proportions of recovered γδ T and Vδ2^+^ T cells among CD3^+^ cells were significantly decreased in the recipients at this time point (Figures 1A,B). Similarly, the absolute numbers of total γδ T cells (Figure 1C) and Vδ2^+^ T cells (Figure 1D) were also lower in recipients than in healthy donors. In contrast, the proportion of Vδ2-negative γδ T cells was not significantly different between the recipients and healthy donors at the same time point (*P* = 0.798, data not shown).

**Table 1:** Clinical characteristics.

	**Recipients**	**Healthy donors**	***P*-value**
Subjects, *n*	35	20	–
Age, years	32 (16-58)	40.5 (19-48)	0.108
**Gender**, ***n*** **(%)**			
Male	15 (42.9)	11 (55)	0.753
Female	20 (57.1)	9 (45)
**Diagnosis**, ***n*** **(%)**			
AML	18 (51.4)	–	–
ALL	10 (28.6)	–	–
CML	1 (2.9)	–	–
MDS	4 (11.4)	–	–
others	2 (5.7)	–	–
Donor type	Related donor	
HLA typing	1–3/6 mismatch	
Stem cell source	BM+PB	
Conditioning regimen	BU+CY+ATG	
GvHD prophylaxis	CSA+MMF+MTX	

*ATG, antithymocyte globulin; AML, acute myeloid leukemia; ALL, acute lymphoblastic leukemia; BM, bone marrow; BU, busulfan; CML, chronic myeloid leukemia; CSA, cyclosporine A; CY, cyclophosphamide; MDS, myelodysplastic syndrome; MMF, mycophenolate mofetil; MTX, methotrexate; PB, peripheral blood*.

Since Vδ2^+^ T cells can be specifically activated and stimulated by aminobisphosphonates, pamidronate in combination with IL-2 was used in the current study to induce the *ex-vivo* expansion of Vδ2^+^ T cells following haploHSCT. As an experimental control, the percentage of γδ T cells was elevated from 11 to 87% in healthy donors by day 14 after stimulation (Figure 1E). Consistent with this finding, the median proportion of Vδ2^+^ T cells increased dramatically from 9 to 80% in this group at 14 days after pamidronate stimulation (Figure 1F). However, we did not observe a significant expansion of Vδ2^+^ T cells from haploHSCT recipients under the same experimental conditions, although the baseline ratios of γδ T/total T cells were similar between the groups (Figures 1E,F). These results indicate that the Vδ2^+^ T cells recovered after haploHSCT are not stimulated by phosphoantigens.

### The expression of BTN3A1 (CD277) on peripheral Vδ2^+^ T cells did not differ between the recipients and healthy subjects

**Figure 2:**
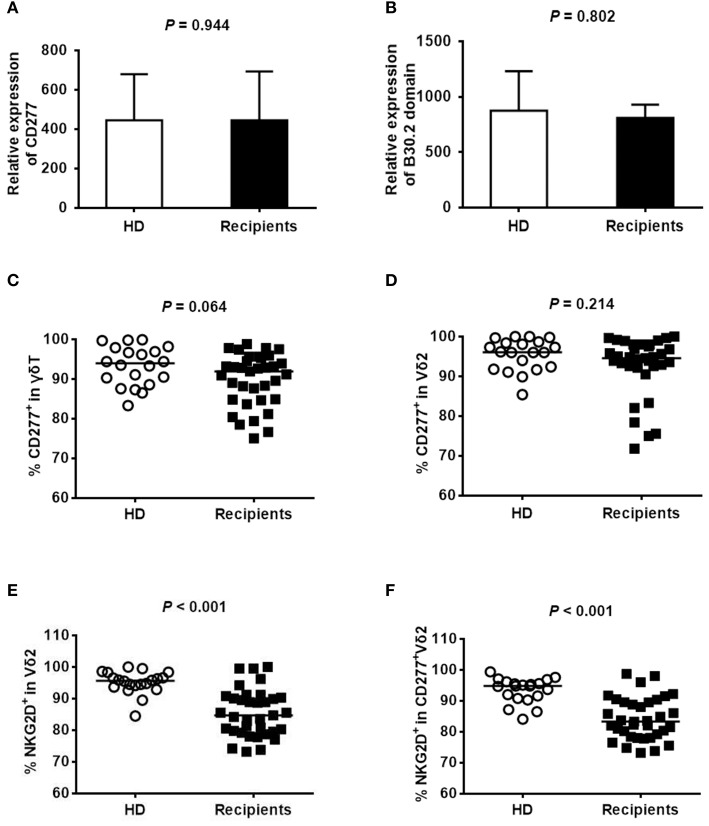
Expression of *CD277* in γδ T cells. The expression levels of *CD277* **(A)** and *B30.2* **(B)** were evaluated by quantitative RT-PCR in purified γδ T cells from healthy donors or recipients ~180 days after haploHSCT (*n* = 5). The level of target gene expression was normalized to the level of the housekeeping gene *ABL*. The percentages of CD277^+^γδ T cells and CD277^+^Vδ2^+^ T cells in healthy donors (*n* = 20) and recipients (*n* = 35) 180 days after haploHSCT are shown in **(C,D)**. The percentages of NKG2D^+^Vδ2 cells and NKG2D^+^CD277^+^Vδ2 cells in healthy donors (*n* = 20) and recipients (*n* = 35) 180 days after haploHSCT are shown in **(E,F)**.

CD277 and its intracellular domain B30.2 are involved in mediating the phosphoantigens-induced proliferation of Vδ2^+^ T cells (19). To explore whether the unsuccessful expansion of recovered Vδ2^+^ T cells that occurs in response to pamidronate was associated with CD277 expression, we analyzed the expression levels of CD277 and B30.2 in γδ T cells from donors and recipients ~180 days after haploHSCT by quantitative RT-PCR and flow cytometry. A representative flow-cytometric analysis of the purity of MACS-isolated γδ T cells from healthy donors and HSCT recipients is shown in Supplemental Figure 1. RT-PCR analysis revealed the similar levels of *CD277* (Figure 2A) and *B30.2* (Figure 2B) transcripts in γδ T cells from recipients and healthy donors (*P* = 0.944 and *P* = 0.802, respectively). The expression of CD277 was also detected by flow cytometry, and the representative images are shown in Supplemental Figure 2. Almost all γδ T and Vδ2^+^ T cells expressed CD277 (Figures 2C,D) and there was no significant difference between the donors and recipients (*P* = 0.064 and *P* = 0.214, respectively). These results indicate that the concentration of CD277 on Vδ2^+^ T cells from recipients is comparable to that on Vδ2^+^ T cells from healthy subjects, which probably does not account for the low levels of Vδ2^+^ T cells after haploHSCT. Almost all of the Vδ2 cells from donors and recipients were Vγ9 positive (99.8 and 99.6%, respectively, data not shown) in the current study. However, the percentage of NKG2D expression in Vδ2 cells (Figure 2E) or CD277^+^Vδ2 cells (Figure 2F) was lower in recipients than in healthy donors (*P* < 0.001, respectively), implying that this receptor might be involved in the unsuccessful *ex-vivo* expansion of Vδ2^+^ T cells after pamidronate treatment.

### The frequencies of DCs subsets were significantly decreased and correlated with the slow recovery of Vδ2^+^ T cells after HaploHSCT

**Figure 3:**
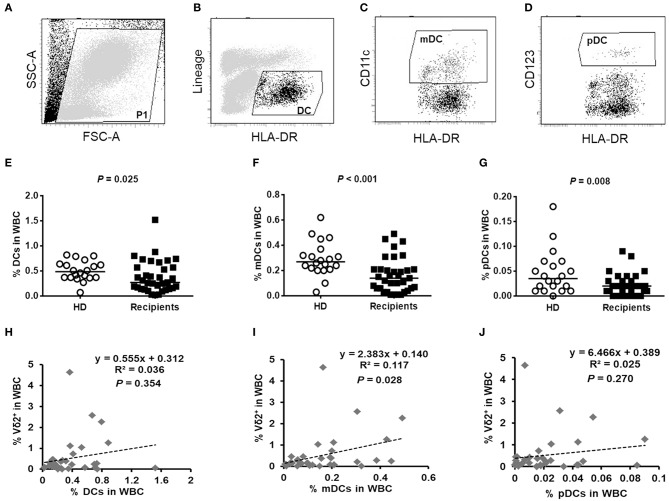
The contents of DCs in healthy donors and haploHSCT recipients and the correlation between DCs and Vδ2^+^ T cell recovery post-haploHSCT. Representative images of the DC gating strategies are shown in **(A–D)**. Peripheral blood DCs were identified as Lin^−^HLA-DR^+^ cells **(B)**, myeloid DCs (mDCs) were identified as Lin^−^HLA-DR^+^CD11c^+^ cells **(C)**, and plasmacytoid DCs (pDCs) were identified as Lin^−^HLA-DR^+^CD123^+^ cells **(D)** by flow cytometry. Comparisons of the proportions of DCs **(E)**, mDCs **(F)**, and pDCs **(G)** in the white blood cells (WBCs) between healthy donors (*n* = 20) and haploHSCT recipients (*n* = 35). Bivariate correlation analyses for the association of DCs **(H)**, mDCs **(I)**, and pDCs **(J)** with the recovery of Vδ2^+^ T cells after haploHSCT. *P*-values are presented on the graphs.

It was reported that primary human DCs are the most potent expander of Vδ2^+^ T cells (30). We next detected the frequencies of DCs and their subsets, including CD123^+^ plasmacytoid DCs (pDCs) and CD11c^+^ myeloid DCs (mDCs), among the white blood cells (WBCs) after haploHSCT. Representative images of the DC gating strategies are shown in Figures 3A–D. Compared with healthy subjects, the recipients had strikingly decreased proportions of DCs (0.49% vs. 0.27%, *P* = 0.025), mDCs (0.27% vs. 0.14%, *P* < 0.001), and pDCs (0.04% vs. 0.02%, *P* = 0.008) in the WBCs ~180 days post-haploHSCT (Figures 3E–G). Furthermore, we investigated whether the recovery levels of Vδ2^+^ T cells were associated with the DC content following transplantation. Bivariate correlation analysis showed that the proportion of mDCs, but not DCs and pDCs, in WBCs was significantly correlated with the recovery of Vδ2^+^ T cells after haploHSCT (Figures 3H–J, *P* = 0.028). The kinetics of the recovery of γδ T-cell and DC subsets at different time points after haploHSCT are summarized in Supplemental Table 1.

### Pamidronate-pretreated DCs efficiently restored the *ex-vivo* expansion of peripheral Vδ2^+^ T cells from HaploHSCT recipients

Given the significant correlation between the levels of mDCs and Vδ2^+^ T cells after haploHSCT, we further explored whether DCs could help trigger the expansion of recovered Vδ2^+^ T cells in the presence of pamidronate. Since DCs are a very small population that accounts for < 1% of human PBMCs and is difficult to maintain in culture, monocyte-derived DCs were generated and pretreated with pamidronate (so-called DCs^pami+^) as described above. The mature DC immunophenotype was detected by flow cytometry and 60–80% of *ex-vivo-*induced DCs exhibited a CD14^−^HLA-DR^+^CD83^+^CD86^high^ phenotype in the current study (data not shown).

**Figure 4:**
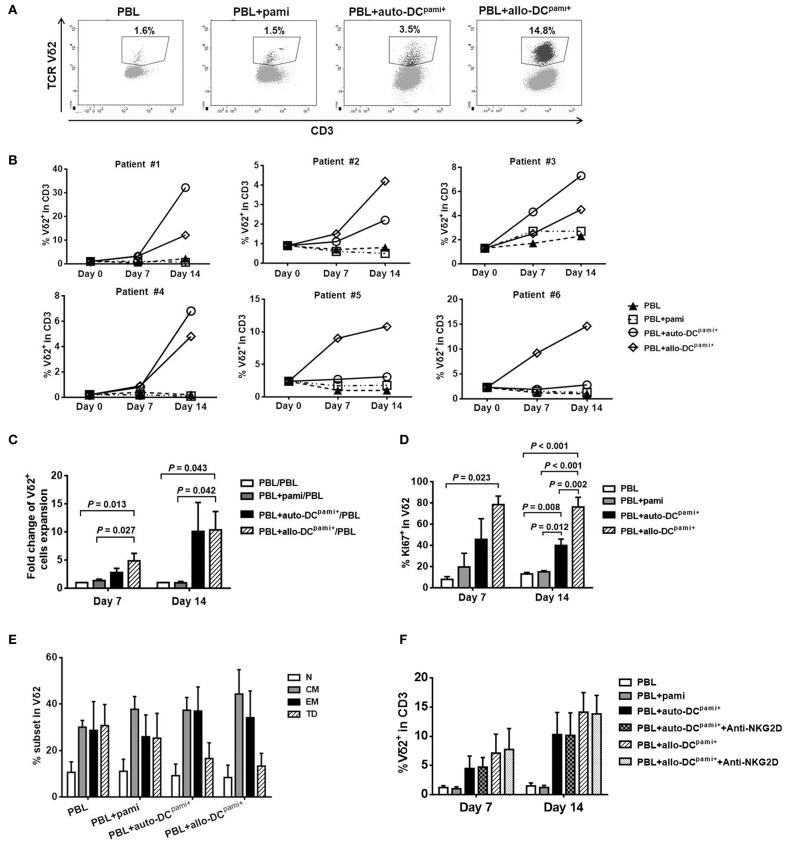
*Ex-vivo* expansion of Vδ2^+^ T cells from haploHSCT recipients after stimulation with pamidronate-pretreated DCs. Peripheral blood lymphocytes (PBLs) from transplant recipients were directly treated with pamidronate or cocultured with monocyte-derived and pamidronate-pretreated autologous or allogeneic (refers to third-party healthy subjects) DCs, which are referred to as auto-DCs^pami+^ or allo-DCs^pami+^ respectively. **(A)** Representative images of flow cytometric analyses of Vδ2^+^ T cell expansion in different groups after 14 days of culture. **(B)** The proportions of Vδ2^+^ T cells in each of 6 recipients after different treatments for 7 and 14 days. **(C)** The average fold changes in Vδ2^+^ T cells expansion in different groups (*n* = 6). **(D)** The average proportions of Ki67^+^Vδ2 cells in different groups (*n* = 3). **(E)** The proportions of Vδ2^+^ T cell fractions with various differentiation statuses in different groups (*n* = 6). **(F)** The proportions of Vδ2^+^ T cells after coculture with auto-DCs^pami+^ or allo-DCs^pami+^, with or without NKG2D blocking antibody (*n* = 3). *P*-values are shown on the graphs.

Then, the PBLs isolated from haploHSCT recipients were directly treated with pamidronate, or cocultured with either autologous or allogeneic third-party donor DCs^pami+^ (auto- or allo-DCs^pami+^). Representative images of flow cytometric analyses of the proportions of Vδ2^+^ T cells in different groups are shown in Figure 4A. The expansion of Vδ2^+^ T cells from 6 individual recipients was detected separately at 7 and 14 days after coculture (Figure 4B). Both auto- and allo-DCs^pami+^ induced a strong expansion of Vδ2^+^ T cells from recipients #1, #2, #3, and #4, while PBLs that were untreated or directly treated with pamidronate did not exhibit obvious Vδ2 cell expansion. In patients #5 and #6, only the allo-DCs^pami+^, but not the auto-DCs^pami+^, efficiently restored the expansion of peripheral Vδ2^+^ T cells from transplant recipients (Figure 4B). Analyses of the fold changes in cell expansion showed that compared with the culture of PBLs alone, coculture with allo-DCs^pami+^ induced 4.86 ± 1.33-fold and 10.35 ± 3.29-fold expansions in Vδ2^+^ T cells at 7 and 14 days (*P* = 0.013 and 0.043, respectively, Figure 4C). Although the auto-DCs^pami+^ from 4 individual recipients exhibited induced Vδ2^+^ T cell expansion, the average fold change did not reach statistical significance compared to the control (Figure 4C). In addition, the expression of Ki67 on Vδ2^+^ T cells, as a marker of cell proliferation, was estimated among the groups. As shown in Figure 4D, the proportions of Ki67-positive Vδ2 cells in cocultures of PBLs with allo-DCs^pami+^ (78.43 ± 7.98% and 76.20 ± 9.05%) were significantly higher than those in PBLs alone (7.83 ± 2.69% and 13.03 ± 1.35%) or PBLs with pamidronate (19.60 ± 12.96% and 15.03 ± 1.18%) at 7 and 14 days, respectively (*P*-values are indicated on the graphs). Of note, compared to auto-DCs^pami+^, allo-DCs^pami+^ induced more Ki67-positive Vδ2 cells at the latter time point (*P* = 0.002, Figure 4D). The effects of pamidronate and DCs^pami+^ on Vδ1 cells were also detected in the same experiments. As shown in Supplemental Figure 3, Vδ1 cells were not expanded by either pamidronate alone or coculture with DCs^pami+^. These results indicate that allo-DCs^pami+^ can trigger a strong proliferation of primary Vδ2^+^ T cells from HSCT recipients.

The differentiation pattern of expanded Vδ2^+^ T cells was detected by staining for CD27 and CD45RO after 14 days of coculture with DCs^pami+^. As shown in Figure 4E, most of the Vδ2^+^ T cells in the PBLs alone group were memory (58.66 ± 12.34%) and terminally differentiated (30.73 ± 9.02%) cells whereas a minor fraction were naïve Vδ2^+^ T cells (10.62 ± 4.51%). After coculture with allo-DCs^pami+^ or auto-DCs^pami+^, the central and effector memory phenotypes of Vδ2^+^ T cells were well-retained compared with those of the control. These results demonstrated that the functional differentiation capacity of Vδ2^+^ T cells was preserved when these cells were expanded by coculture with DCs^pami+^.

Given that the expression of NKG2D in Vδ2 cells was significantly lower in recipients than in healthy donors, we next determined whether NKG2D is required for the DC^pami+^-stimulated expansion of recipient Vδ2 cells. As shown in Figure 4F, treatment with a NKG2D blocking antibody did not inhibit the expansion of Vδ2 cells compared with that of the control under the same experimental condition shown in Figure 4B. This result is consistent with the results of a previous study that showed that blocking of the NKG2D receptor did not affect the proliferation of Vδ2^+^ T cells (31).

### Pamidronate-pretreated DCs promote the activation and cytotoxic cytokine production of primary Vδ2^+^ T cells from HaploHSCT recipients

**Figure 5:**
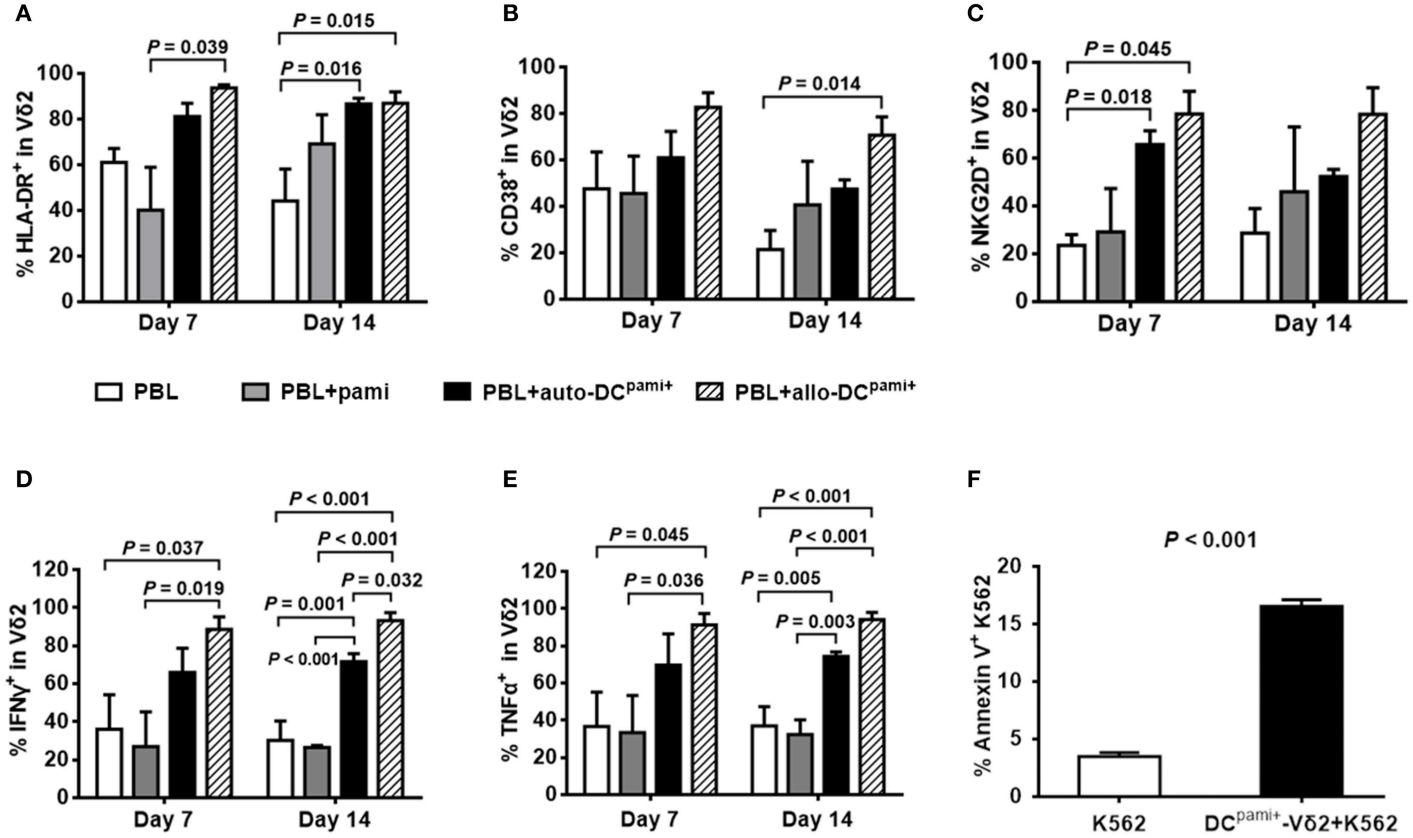
The activation and cytokine production of Vδ2^+^ T cells from haploHSCT recipients after stimulation with pamidronate-pretreated DCs. The average proportions of HLA-DR^+^ **(A)**, CD38^+^ **(B)**, NKG2D^+^ **(C)**, IFN-γ^+^ **(D)**, and TNF-α^+^ **(E)** Vδ2 cells were compared among different treatment groups (*n* = 3). **(F)** Cytotoxicity assay of DCs^pami+^-expanded Vδ2^+^ T cells with K562 cells. *P*-values are shown on the graphs.

Next, the activation status of the expanded Vδ2^+^ T cells was detected. Compared with the culture of PBLs alone, HLA-DR-positive (Figure 5A) and CD38-positive (Figure 5B) Vδ2^+^ T cells were markedly increased upon coculture with different DCs^pami+^ (*P*-values are indicated on the graphs), especially in the case of coculture with allo-DCs^pami+^ at 14 days (*P* = 0.015 and *P* = 0.014, respectively). Compared with that of the control groups, the expression of the functional cytotoxic receptor NKG2D was also significantly elevated in Vδ2^+^ T cells at 7 days after co-culture with allo-DCs^pami+^ (*P* = 0.045, Figure 5C). Upon phorbol myristate acetate (PMA) stimulation, the proportions of IFN-γ- and TNF-α-expressing Vδ2 cells were significantly increased by coculture of PBLs with allo-DCs^pami+^ compared with those of the PBLs groups at 7 and 14 days (*P* values are indicated on the graphs, Figures 5D,E).

Furthermore, the cytotoxic activity of DC^pami+^-induced Vδ2^+^ T cells was evaluated in comparison to that of the K562 leukemia cell line. As shown in Figure 5F, in K562 cells, the apoptotic population was increased from 3.8 to 16.5% after coculture with DC-induced Vδ2 cells. Taken together, these results suggest that allo-DCs^pami+^ not only stimulate the activation and expansion but also enhance the cytotoxic capacity of the Vδ2^+^ T cells reconstituted after allogeneic HSCT.

## Discussion

As innate-like effector lymphocytes, γδ T cells play a critical role in immunosurveillance against infections and tumors (14, 32, 33). Due to their rapid responsiveness to various antigens and their MHC-independent cytotoxicity, γδ T cells have received increasing attentions in the context of HSCT (6, 7, 34, 35). Several studies reported favorable outcomes of haploHSCT with the depletion of CD19^+^ B cells and αβ^+^ T cells in grafts in which the functional γδ T cells were preserved and recovered promptly after transplantation (36–38). However, the role of γδ T subsets in the context of allogeneic HSCT has been studied less thoroughly. We previously found that the recovery of Vδ2^+^ T cells was continuously delayed at an early stage after unmanipulated haploHSCT, which was significantly correlated with the occurrence of EBV reactivation (12). Thus, how to promote the reconstitution of Vδ2^+^ T cells is of interest and impacts the clinical benefits of hematopoietic transplantation. The specific activation and expansion properties of Vδ2^+^ T cells in response to stimulation with aminobisphosphonates makes this special T-cell subpopulation a promising candidate for adoptive immunotherapy for both solid and hematopoietic malignancies (39). Unfortunately, as shown here, direct stimulation with pamidronate did not trigger a successful expansion of the Vδ2^+^ T cells recovered ~180 days after haploHSCT, although the stimulation worked well on the Vδ2 cells from healthy subjects under the same experimental condition. The protocols of PBMC isolation from donors and recipients were identical, and the fresh PBMCs were immediately cultured without storage. It can be speculated that recipient Vδ2 cells are likely affected by the use of immunosuppressive drugs peri- and post-HSCT prophylaxis and treatment. Another concern is about the influence of γδ T composition in grafts. We previously demonstrated a significant correlation of the donor Vδ2 component with recipient Vδ2 cells at 30 days, but not 60, 90, and 180 days, after haploHSCT (29). This observation also suggests the influence of other factors, such as DCs, on the recovery of Vδ2^+^ T cells in the context of allogeneic HSCT.

The activation of Vδ2^+^ T cells by phosphoantigens relies on the accumulation of IPP in professional antigen presenting cells, especially in DCs. Previous studies have reported functional interactions between DCs and Vδ2^+^ T cells (23, 40). Treatment with zoledronic acid enhanced the ability of DCs to activate autologous Vδ2^+^ T cells from healthy individuals (20) and patients with advanced cancer (21). However, the investigations mentioned above were all performed in non-transplant models, and there is no evidences available concerning the influence of DCs on γδ T cells in the setting of HSCT. In the present study, we found that the recovery of DC subsets was significantly decreased and the frequency of mDCs was correlated with Vδ2 cells recovery after haploHSCT. Coculture of recipient PBLs with pamidronate-pretreated autologous or allogeneic DCs induced a strong expansion of primary Vδ2 cells in PBLs. The successful expansions triggered by pamidronate-pretreated DCs, rather than by direct stimulation with pamidronate, could be explained by the idea that *ex-vivo* induction promoted the functional maturation of DCs, and pretreatment with pamidronate led to the accumulation of a sufficient concentration of IPP in DCs. More importantly, our results showed that the effector memory phenotypes were retained and the IFN-γ- and TNF-α-production capacities were enhanced in the expanded Vδ2^+^ T cells. These results imply that pamidronate-pretreated DCs are a promising stimulator for boosting Vδ2^+^ T cells recovery after HSCT. The differential abilities of pamidronate-pretreated autologous DCs and third-party DCs to activate recipient Vδ2^+^ T cell expansion were also investigated in the current study. To our knowledge, this is the first report of such a side-by-side comparison. As shown here, DCs were induced *ex vivo* to undergo functional maturation from recipients' CD14^+^ cells, but these autologous DCs^pami+^ exhibited a lower efficiency than the allogeneic DCs^pami+^ (from the third-party donors) in mediating the expansion of recipient Vδ2^+^ T cells. Given that the successful stimulation of Vδ2 cells by phosphoantigen-pretreated autologous DCs has been reported in non-transplant patients with hematopoietic malignancy (22), the different effects of the recipients and third-party donor DCs may be attributed to the immunosuppressive influences on the recipients peri- and post-haploHSCT. Indeed, it has been reported that antithymocyte globulin (ATG) interfers with various aspects of DC functions and suppresses the T-cell proliferation induced by mature DCs (41, 42). Previous studies reported the distribution, maturation and migration of DC subsets in mucosal and secondary lymphoid tissues where they functioned in tissue surveillance (43, 44). The differences in maturation and antigen uptake and presentation abilities between DCs from recipients and healthy subjects warrant further investigation. Regardless, our findings suggest an alternative strategy that utilizes third-party donor DCs to increase Vδ2^+^ T cells concentrations in immunodepressed patients after allogeneic HSCT.

Interestingly, varying expansion efficiencies of Vδ2^+^ T cells from different haploHSCT recipients were also found after stimulation with third-party donor DCs^pami+^, which were generally lower than the results reported in healthy subjects and non-transplant patients (20, 21). One explanation could be that the baseline proportions of Vδ2^+^ T cells in recipients varied and were generally lower than those of healthy subjects and non-transplant patients. Previous studies reported that the *ex vivo* responsiveness to zoledronate of Vδ2^+^ T cells with lower frequencies was significantly diminished in patients with solid tumors (45, 46). In parallel with these findings, our unpublished data showed that the recovery levels of Vδ2^+^ T cells after HSCT from a HLA-matched related donor were higher than those after haploHSCT, and these Vδ2^+^ T cells were activated by *ex vivo* treatment with pamidronate although the expansion efficiency was lower than that of Vδ2^+^ T cells from healthy subjects. Several studies have reported that the quantity and functions of γδ T cells were affected in immunocompromised patients with solid cancer (47) and HIV infection (48). Recipients who undergo haploHSCT experience more intense immunosuppressive treatment for conditioning and GVHD prophylaxis. Commonly used immunosuppressive drugs, such as ATG, mycophenolate mofetil, and cyclosporin A, have been reported to inhibit T cell proliferation and reduce cell viability (49, 50). The exact influences of these immunosuppressive drugs on the recovery of γδ T cells are still unknown and await further investigations. Next-generation sequencing analysis may help us comprehensively understand the molecular mechanism of irresponsive Vδ2 cells after allogeneic HSCT.

In summary, the present study highlighted the correlation between DCs content and Vδ2^+^ T cell recovery after allogeneic HSCT. Furthermore, we showed that pamidronate-pretreated third-party DCs efficiently restored the *ex-vivo* expansion of peripheral Vδ2^+^ T cells from haploHSCT recipients. The expanded Vδ2^+^ T cells retained their memory features and exhibited an enhanced cytotoxic cytokines-production capacity. These results not only emphasize the critical role of DCs for Vδ2^+^ T cells recovery but also provide a promising strategy for immunotherapy that takes advantage of the effector functions of Vδ2^+^ T cells in patients who undergo hematopoietic transplantations.

## Ethics statement

All included subjects provided with written informed consent for the use of biospecimens for research purposes in accordance with the Declaration of Helsinki. The study was approved by the Ethics Committee of the Peking University Institute of Hematology and carried out in accordance with the approved guideline Use of experimental animals and human subjects.

## Author contributions

XW and JL designed the research, prepared and analyzed data, and wrote the manuscript. HG, X-DM, and TH prepared data. L-PX and X-HZ interpreted data. X-JH supervised research and revised the manuscript. All authors have read and approved the final manuscript.

### Conflict of interest statement

The authors declare that the research was conducted in the absence of any commercial or financial relationships that could be construed as a potential conflict of interest.
