# Supplementary material for: Dendritic Cells Are Critical for the Activation and Expansion of Vδ2+ T Cells After Allogeneic Hematopoietic Transplantation
Source: Front Immunol. 2018 Nov 1;9:2528. doi: 10.3389/fimmu.2018.02528 (PMC6221956; doi:10.3389/fimmu.2018.02528)
Supplement: Supplementary file 1 [file Data_Sheet_1.PDF]

Supplemental Table 1. The percentage of recovered immune cells after haploHSCT

	CD3/WBC	γδT/CD3	Vδ2^{+}/CD3	DC/WBC
Day 30	2.1 (0.1-15)	6.1 (1.2-59)	3 (0-30)	0.1 (0-1.1)
Day 90	21 (1.4-57)	4.7 (0.9-30)	0.6 (0-4.9)	0.1 (0-1.2)
Day 180	34 (5.2-81)	3.7 (0.4-18)	0.6 (0.1-11)	0.6 (0.1-2.2)
Day 360	31 (0.6-75)	5.3 (0.4-24)	0.5 (0-3.5)	1.2 (0.1-4.0)

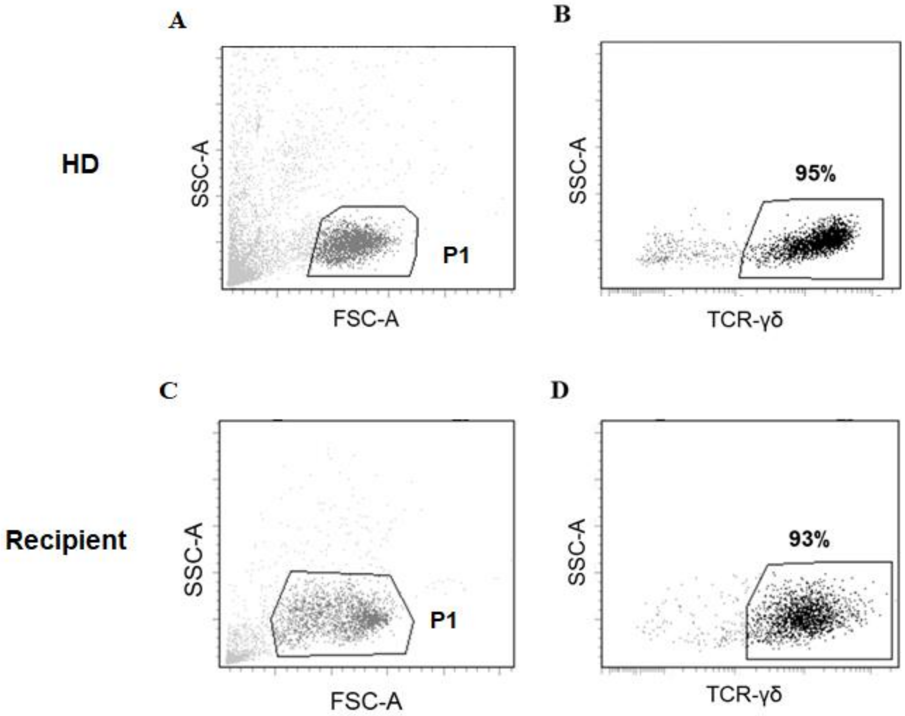

Supplemental Figure 1. Representative flow-cytometry analysis for the purity of MACS-isolated γδ T cells.

(A, B) The proportion of γδ T cells isolated from a healthy donor (HD); (C, D) The proportion of γδ T cells isolated from a haploHSCT recipient.

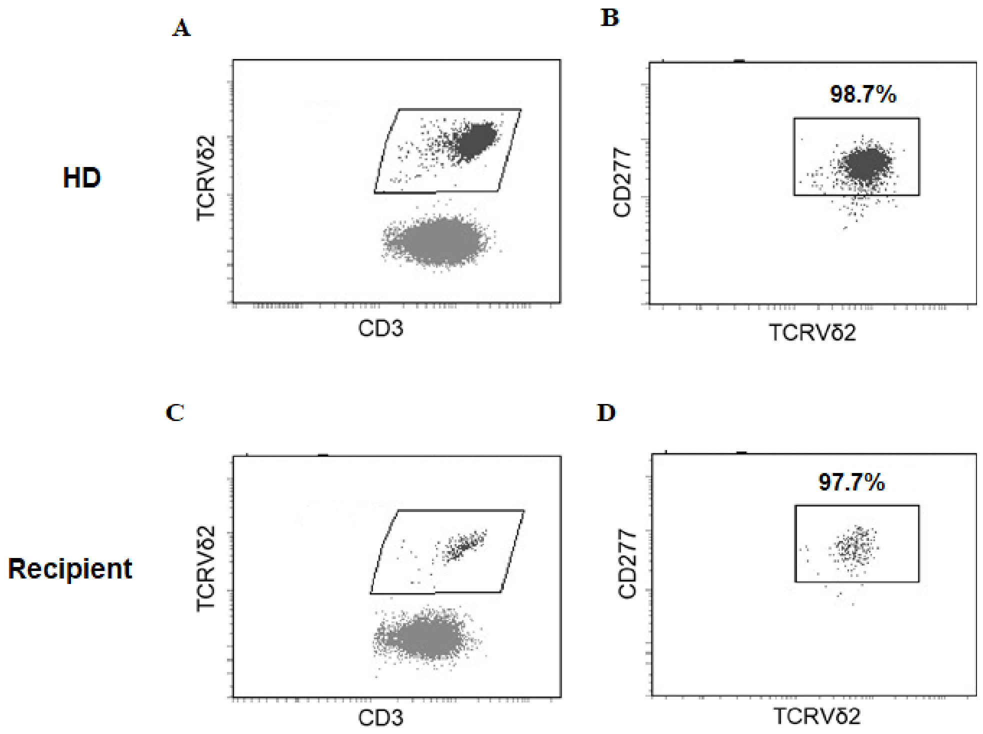

Supplemental Figure 2. Representative images of CD277^{+}Vδ2 cells by flow cytometry.

(A, B) The expression of CD277 in Vδ2 cells from a healthy donor (HD); (C, D) The expression of CD277 in Vδ2 cells from a haploHSCT recipient.

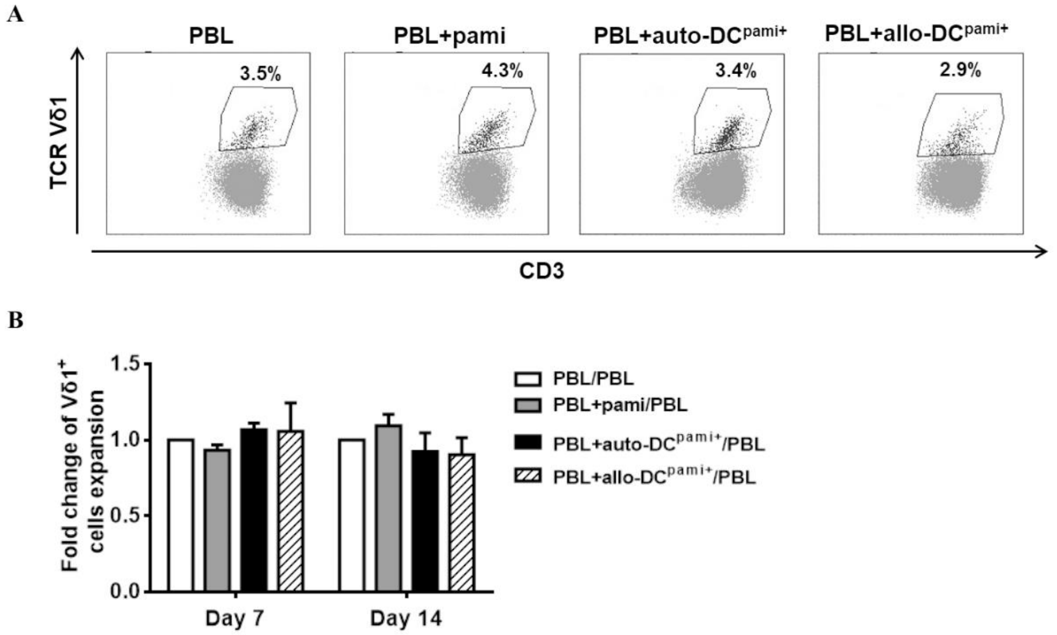

Supplemental Figure 3. Ex-vivo expansion of Vδ1^{+} T cells from haploHSCT recipients after stimulation with pamidronate-pretreated DCs.

Peripheral blood lymphocytes (PBLs) from transplant recipients were directly treated with pamidronate or cocultured with monocyte-derived and pamidronate-pretreated autologous or allogeneic (refers to third-party healthy subjects) DCs, which are referred to as auto-DCs^{pami+} or allo-DCs^{pami+} respectively. (A) Representative images of flow cytometric analyses of Vδ1^{+} T cell expansion in different groups after 14 days of culture. (B) The average fold changes in Vδ1^{+} T cells expansion in different groups (n=3).
